# Factors Associated with Nonachievement of Target Blood Pressure in Patients on Antithrombotic Therapy: A Real-World Study

**DOI:** 10.31662/jmaj.2021-0146

**Published:** 2021-12-15

**Authors:** Naoyuki Odaguchi, Atsushi Sakima, Tomohiro Yara, Isao Shiroma

**Affiliations:** 1 Blood Purification Center, Keiaikai Chibana Clinic, Okinawa, Japan; 2 Health Administration Center, University of the Ryukyus, Okinawa, Japan; 3 Division of Internal Medicine, Keiaikai Chibana Clinic, Okinawa, Japan

**Keywords:** blood pressure, antiplatelet therapy, anticoagulant therapy, antihypertensive therapy, obesity, cardiovascular comorbidity, clinical practice

## Abstract

**Introduction:**

The current guidelines for managing hypertension recommend strict blood pressure (BP) control to prevent bleeding complications in patients with hypertension on antithrombotic therapy. However, the target BP value of <130/80 mmHg is achieved in a small proportion of these patients. This study aimed to examine the factors associated with nonachievement of target BP value (≥130/80 mmHg) in patients on antithrombotic therapy.

**Methods:**

This retrospective study was conducted at an outpatient clinic in 2018. Clinical parameters were obtained from the center’s electronic medical database. Office BP was measured once in the sitting position. A target BP value of <130/80 mmHg was defined according to the Japanese Society of Hypertension Guidelines for the Management of Hypertension 2019.

**Results:**

Of the 26,803 outpatients who had scheduled visits during this time, 2,427 received antithrombotic therapy. Patients with chronic kidney disease stage 5 or on hemodialysis and those with missing data on body mass index were excluded from the study; eventually, 2,201 outpatients met the inclusion criteria. BP values of <140/90 mmHg were observed in 59.2% of these outpatients; however, only 30.6% displayed the target BP value of <130/80 mmHg. Univariate and multivariate logistic regression analyses indicated that male gender and obesity significantly correlated with nonachievement of the target BP (≥130/80 mmHg). However, heart failure and ischemic heart disease were negatively but significantly related to nonachievement of the target BP.

**Conclusions:**

The target BP value was achieved in only a small proportion of the patients treated with antithrombotic drugs. In patients on antithrombotic therapy, obesity appeared to be a modifiable risk factor, whereas cardiovascular comorbidities, such as heart failure, were negative factors contributing to nonachievement of the target BP.

## Introduction

Although antithrombotic therapy is widely prescribed for the secondary prevention of ischemic stroke, ischemic heart disease, and other cardiovascular diseases, it has been associated with a modest increase in hemorrhagic complications ^(1), (2), (3)^. Observational studies ^(4), (5), (6), (7)^, post hoc analyses of randomized clinical trials (RCTs) ^(8), (9), (10)^, and phase III clinical trials of non-vitamin K antagonist oral anticoagulants ^(11), (12)^ have demonstrated a strong association between blood pressure (BP) levels and the hemorrhagic complications of antithrombotic therapy. However, the target BP value to prevent the hemorrhagic complications of antithrombotic therapy remains to be determined.

Among the recently updated major hypertension guidelines ^(13), (14), (15)^, the Japanese Society of Hypertension Guidelines for the Management of Hypertension 2019 (JSH 2019) recommend that BP should be carefully controlled in patients with hypertension on antithrombotic therapy, with a target BP value of <130/80 mmHg, despite the insufficient evidence for target BP values that prevent bleeding complications during antithrombotic therapy ^(15)^. We recently demonstrated that most patients with hypertension on antithrombotic therapy in real-world practice exhibit a BP value of <140/90 mmHg, while the target BP value of <130/80 mmHg is achieved only in a small proportion of these patients ^(16)^. However, other previous studies on the association between BP and the hemorrhagic complications of antithrombotic therapy have included not only patients with hypertension but also those without ^(4), (5), (6), (7), (8), (9), (10), (11), (12), (17), (18)^. Accordingly, this study aimed to investigate the attainment status of target BP (130/80 mmHg) and the factors associated with nonachievement of target BP in patients receiving antithrombotic therapy in real-world practice.

## Materials and Methods

This study was designed in accordance with the principles of the Declaration of Helsinki and was approved by the human research ethics committee of Keiaikai (Approval No. 2021001). Because this was a retrospective study, written informed consent could not be obtained from each patient. However, according to the Ethical Guidelines for Clinical Research issued by The Japanese Ministry of Health, Labour and Welfare, all relevant details regarding the objectives and procedure of this study were published on the official homepage of the authors’ hospital to allow patients to refuse participation in the study if they so wished.

### Patients

Clinical data from outpatients attending the Keiaikai Chibana Clinic between January 1, 2018 and December 31, 2018 were obtained from the center’s electronic medical database. Patients who met the following criteria were recruited: (1) age of ≥20 years, (2) at least one regular scheduled visit to the clinic, (3) at least 1 month of regular follow-up, and (4) an office BP measurement. Those with chronic kidney disease (CKD) stage 5, on hemodialysis, and with missing data on the body mass index (BMI) were excluded from the study.

### Data extraction

The extracted data included age, gender, office BP, height, body weight, BMI, cardiovascular risk factors, past history of cardiovascular disease, comorbidities, prescriptions, and routine laboratory tests. Office BP was measured once in the sitting position using an aneroid or electric automatic sphygmomanometer at the first scheduled visit. Hypertension was defined as systolic BP (SBP) of ≥140 mmHg, diastolic BP (DBP) of ≥90 mmHg, or ongoing treatment with antihypertensive drugs. The office BP value was classified according to the JSH 2019 guidelines as follows: normal BP, SBP of <120 mmHg and DBP of <80 mmHg; high-normal BP, SBP of 120-129 mmHg and DBP of <80 mmHg; elevated BP, SBP of 130-139 mmHg and/or DBP of 80-89 mmHg; grade I hypertension, SBP of 140-159 mmHg and/or DBP of 90-99 mmHg; grade II hypertension, SBP of 160-179 mmHg and/or DBP of 100-109 mmHg; and grade III hypertension, SBP of ≥180 mmHg and/or DBP of ≥110 mmHg ^(15)^. Obesity was defined as BMI of ≥25 kg/m^2^; diabetes mellitus as fasting blood glucose (BG) of ≥126 mg/dL, casual BG of ≥200 mg/dL, hemoglobin A1c of ≥6.5%, or using antidiabetic drugs; dyslipidemia as low-density lipoprotein cholesterol of ≥140 mg/dL, triglycerides of ≥150 mg/dL, high-density lipoprotein cholesterol of <40 mg/dL, or using antilipidemic drugs; and CKD as an estimated glomerular filtration rate of <60 mL/min/1.73 m^2^ or proteinuria by urine dipstick test (≥1+). Atrial fibrillation (AF) was recorded if documented any time on 12-lead electrocardiograms or Holter monitors. The definitions were taken from the International Statistical Classification of Diseases and Related Health Problems 10^th^ revision (ICD-10) codes as follows: hypertension (I10-I15), diabetes mellitus (E10-E14), dyslipidemia (E78), cerebrovascular disease (I60-I69), ischemic heart disease (I20-I25), heart failure (I50), CKD (N18), and AF (I48).

Diagnoses of cerebrovascular disease, ischemic heart disease, and heart failure were also extracted from the database. Cardiovascular risk was then categorized according to the JSH 2019 guidelines as follows: category I, patients without any factors affecting the prognosis other than BP; category II, having at least one factor except age of ≥65 years, male gender, dyslipidemia, and current smoking; category III, having at least one factor other than a history of cardiovascular diseases, nonvalvular AF, diabetes mellitus, and proteinuria positive CKD, or ≥3 of the category II risk factors ^(15)^.

### Outcomes

The primary outcomes of interest were the rate of attainment of the target BP value of <130/80 mmHg and factors associated with nonachievement of target BP in patients on antithrombotic therapy. The secondary outcomes were the status of BP distribution and cardiovascular risk categorization in this population.

### Statistical analysis

Continuous variables are expressed as mean ± standard deviation. Categorical variables are presented as frequencies and percentages. Categorical variables were analyzed using Chi-square test. The odds ratios (ORs) for nonachievement of target BP were calculated using univariate and multivariate logistic regression analyses. The explanatory variables for the multivariate analysis were also adopted from well-known cardiovascular risk factors and comorbidities, such as cerebrovascular disease, ischemic heart disease, heart failure, CKD, and AF. Additionally, the ORs for nonachievement of target BP were also calculated in patients with or without AF. Antihypertensive drug use was not included as an explanatory variable in the multivariate analysis to avoid multicollinearity. We used JMP version 13.0.0 (SAS Institute, Cary, NC, USA) for statistical analysis. Two-sided p-values of <0.05 were considered statistically significant.

## Results

### Clinical characteristics

Of the 26,803 screened subjects, 2,427 received antithrombotic therapy. Following the exclusion of outpatients with CKD stage 5 (n = 17), patients on hemodialysis (n = 12), and patients with missing data on BMI (n = 197), the remaining 2,201 patients met the inclusion criteria. Table 1 shows the demographic characteristics, prevalence of comorbidities, classes of antihypertensive drugs, and classes of antithrombotic drugs for all patients. Most patients had complications of hypertension, dyslipidemia, and obesity. Furthermore, the majority of patients were on antihypertensive therapy, with the most frequently prescribed drugs being calcium channel blockers, followed by angiotensin II receptor blockers, and then beta-blockers. The most commonly prescribed antithrombotic agents were acetylsalicylic acid, followed by P2Y12 receptor inhibitors, and then warfarin.

**Table 1.** Clinical Characteristics of Patients on Antithrombotic Therapy.

Clinical parameters	Mean ± SD, percentage, or number
Number	2201
Age (years)	74.1 ± 11.1
Male gender (%)	59.5
SBP (mmHg)	134.9 ± 17.1
DBP (mmHg)	74.1 ± 12.9
BMI (kg/m^2^)	25.7 ± 4.3
eGFR (mL/min/1.73 m^2^)	66.3 ± 22.0
Obesity (%)	56.2
Hypertension (%)	85.2
Diabetes mellitus (%)	47.8
Dyslipidemia (%)	71.5
Cerebrovascular disease (%)	46.0
Ischemic heart disease (%)	38.0
Heart failure (%)	19.4
Atrial fibrillation (%)	17.3
Chronic kidney disease (%)	43.8
Antihypertensive therapy (%)	79.7
ACE inhibitor (%)	14.4
ARB (%)	41.9
CCB (%)	52.2
Beta-blocker (%)	21.7
Diuretics (%)	16.5
Antithrombotic therapy (%)	100
Acetylsalicylic acid (%)	68.1
P2Y12 receptor inhibitor (%)	18.0
PDE-III inhibitor (%)	5.7
Warfarin (%)	13.3
Direct factor Xa inhibitor (%)	6.5
Direct thrombin inhibitor (%)	2.8

Abbreviations: ACE, angiotensin-converting enzyme; ARB, angiotensin II receptor blocker; BMI, body mass index; CCB, calcium channel blocker; DBP, diastolic blood pressure; eGFR, estimated glomerular filtration rate; PDE, phosphodiesterase; SBP, systolic blood pressure; SD, standard deviation

### BP distribution, status of BP control, and cardiovascular risk profile

**Figure 1.**
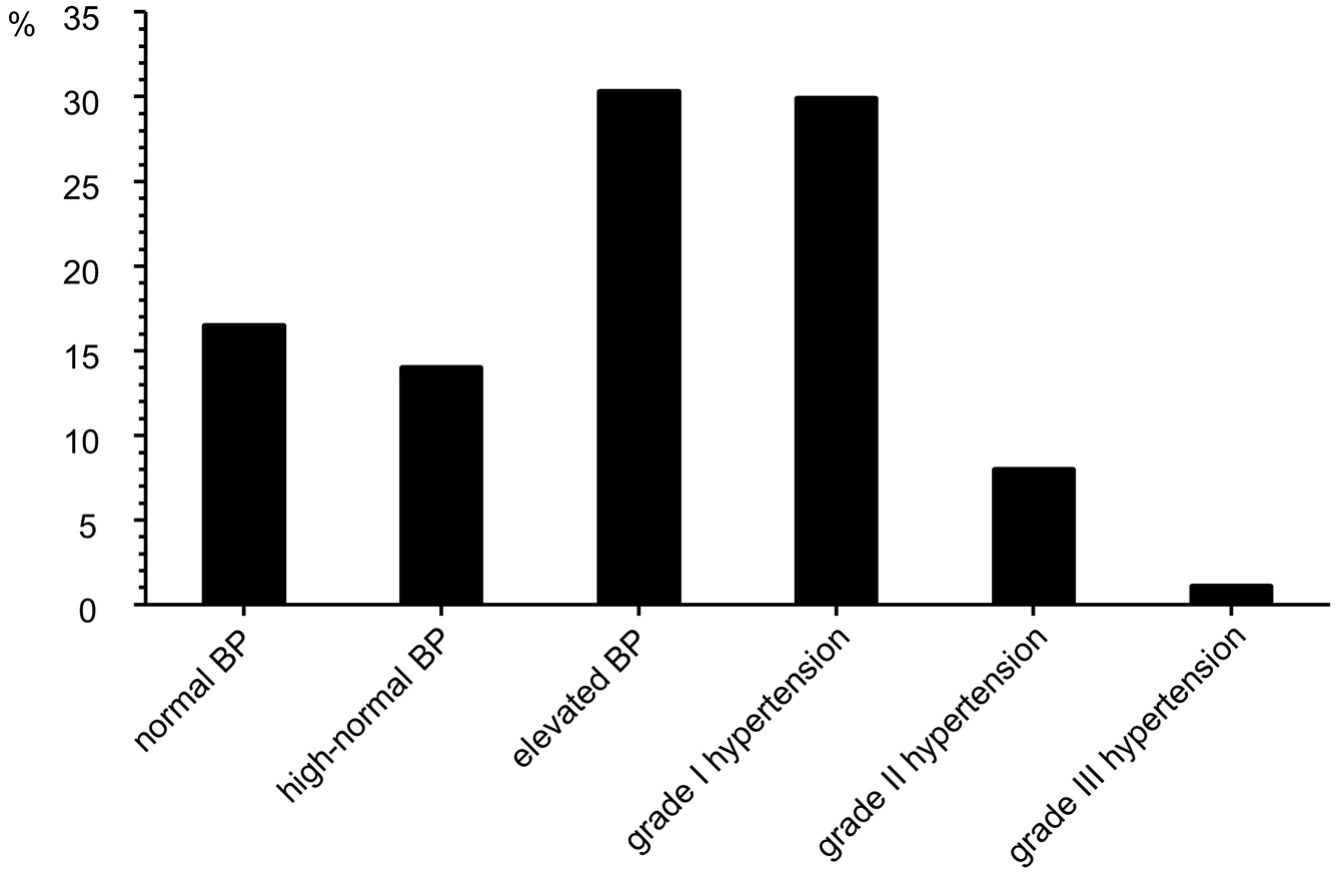
Blood pressure distribution in patients on antithrombotic therapy Blood pressure distribution was described according to the JSH 2019 guidelines. In this population, 16.5%, 14.0%, 30.3%, 29.9%, 8.0%, and 1.1% had normal BP, high-normal BP, elevated BP, grade I hypertension, grade II hypertension, and grade III hypertension, respectively.

Figure 1 shows the distribution of the BP in this population. As illustrated, 16.5%, 14.0%, 30.3%, 29.9%, 8.0%, and 1.1% had normal BP, high-normal BP, elevated BP, grade I hypertension, grade II hypertension, and grade III hypertension, respectively. A BP value of <140/90 mmHg was attained in 59.2% of this population, and only 30.6% of the participants displayed the target BP value of <130/80 mmHg. Table 2 shows the achievement rates of the target BP value of <130/80 mmHg and the clinical characteristics in patients on antithrombotic therapy. The achievement rates of the target BP of patients of the male gender, obese, and dyslipidemia were significantly lower than those of patients of the female gender, nonobese, and without dyslipidemia, respectively. Meanwhile, the achievement rates of the target BP of patients of the age of ≥75 years, with heart failure, and with ischemic heart disease were significantly higher than those of patients of the age of <75 years, without heart failure, and with nonischemic heart disease, respectively. The cardiovascular risk status according to the JSH 2019 ^(15)^ revealed that 0.2%, 5.2%, and 94.6% of the patients on antithrombotic therapy were in risk categories 1, 2, and 3, respectively.

**Table 2.** Achievement Rates of Target Blood Pressure Value of <130/80 mmHg in Patients on Antithrombotic Therapy.

Variable	Achievement of the target blood pressure value		
	Yes, n (%)	No, n (%)	P-value
Age			0.031
<75 years	279 (28.2)	709 (71.8)
≥75 years	394 (32.5)	819 (67.5)
Gender			0.008
Male	372 (28.4)	937 (71.6)
Female	301 (33.7)	591 (66.3)
Obesity			<0.001
Yes	317 (25.6)	920 (74.4)
No	356 (36.9)	608 (63.1)
Diabetes mellitus			0.783
Yes	319 (30.3)	734 (69.7)
No	354 (30.8)	794 (69.2)
Dyslipidemia			0.049
Yes	462 (29.4)	1112 (70.7)
No	211 (33.7)	416 (66.4)
Cerebrovascular disease			0.693
Yes	314 (31.0)	699 (69.0)
No	359 (30.2)	829 (69.8)
Ischemic heart disease			0.021
Yes	280 (33.5)	556 (66.5)
No	393 (28.8)	972 (71.2)
Heart failure			<0.001
Yes	184 (43.2)	242 (56.8)
No	489 (27.6)	1286 (72.5)
Atrial fibrillation			0.163
Yes	128 (33.6)	253 (66.4)
No	545 (30.0)	1275 (70.1)
Chronic kidney disease	
Yes	300 (31.2)	663 (68.9)	0.605
No	373 (30.1)	865 (69.9)	

The JSH 2019 proposes that BP control with a target SBP of <130 mmHg should be performed in patients with AF ^(15)^. Therefore, we conducted a subgroup analysis of the status of BP control in 381 patients with AF as a complication. A mean SBP of 131.0 ± 18.5 mmHg and a mean DBP of 76.0 ± 14.0 mmHg were observed in patients with AF on antithrombotic therapy, of whom 42.8% achieved a target SBP value of <130 mmHg.

### Factors associated with nonachievement of target BP

Univariate logistic regression models indicated that male gender (OR = 1.283, 95% confidence interval [CI] = 1.068-1.541, P = 0.008), obesity (OR = 1.699, 95% CI = 1.416-2.041, P < 0.001), and dyslipidemia (OR = 1.221, 95% CI = 1.000-1.487, P = 0.049) were positively associated with nonachievement of the target BP (≥130/80 mmHg) in patients on antithrombotic therapy. Heart failure (OR = 0.500, 95% CI = 0.402-0.622, P < 0.001), ischemic heart disease (OR = 0.803, 95% CI = 0.667-0.967, P = 0.021), and age of ≥75 years (OR = 0.818, 95% CI = 0.681-0.982, P = 0.031) were negatively associated with nonachievement of the target BP (Table 3). Multivariate logistic regression models also demonstrated that male gender (OR = 1.263, 95% CI = 1.035-1.542, P = 0.022) and obesity (OR = 1.703, 95% CI = 1.399-2.075, P < 0.001) were positively associated with nonachievement of the target BP (≥130/80 mmHg) in patients on antithrombotic therapy. However, heart failure (OR = 0.493, 95% CI = 0.385-0.631, P < 0.001), ischemic heart disease (OR = 0.737, 95% CI = 0.587-0.927, P = 0.009), and cerebrovascular disease (OR = 0.748, 95% CI = 0.600-0.932, P = 0.010) were negatively associated with nonachievement of the target BP (Table 3). Additionally, we conducted logistic regression analyses of factors associated with nonachievement of target BP in patients with and without AF. In patients with AF, univariate and multivariate logistic regression models demonstrated that obesity was positively associated with nonachievement of the target BP, whereas heart failure was negatively associated with nonachievement of the target BP (Table 4). In patients without AF, univariate and multivariate logistic regression models also demonstrated that obesity was positively associated with nonachievement of the target BP, whereas heart failure and ischemic heart disease were negatively associated with nonachievement of the target BP (Table 5).

**Table 3.** Logistic Regression Model of Factors Associated with Nonachievement of Target Blood Pressure Control (≥130/80 mmHg) in Patients on Antithrombotic Therapy.

	Univariate model			Multivariate model		
Variable	Odds ratio	95% CI	P-value	Odds ratio	95% CI	P-value
Age, ≥75 years	0.818	0.681-0.982	0.031	0.953	0.774-1.174	0.651
Male gender	1.283	1.068-1.541	0.008	1.263	1.035-1.542	0.022
Obesity	1.699	1.416-2.041	<0.001	1.703	1.399-2.075	<0.001
Diabetes mellitus	1.026	0.856-1.230	0.783	0.917	0.751-1.121	0.398
Dyslipidemia	1.221	1.000-1.487	0.049	1.241	0.987-1.558	0.064
Cerebrovascular disease	0.964	0.803-1.156	0.693	0.748	0.600-0.932	0.010
Ischemic heart disease	0.803	0.667-0.967	0.021	0.737	0.587-0.927	0.009
Heart failure	0.500	0.402-0.622	<0.001	0.493	0.385-0.631	<0.001
Atrial fibrillation	0.845	0.669-1.071	0.163	0.916	0.696-1.208	0.531
eGFR (mL/min/1.73 m^2^)	1.004	1.000-1.008	0.081	1.002	0.997-1.007	0.400

Abbreviations: CI, confidence interval; eGFR, estimated glomerular filtration rate

**Table 4.** Logistic Regression Model of Factors Associated with Nonachievement of Target Blood Pressure Control (≥130/80 mmHg) in Patients with Atrial Fibrillation on Antithrombotic Therapy.

	Univariate model			Multivariate model		
Variable	Odds ratio	95% CI	P-value	Odds ratio	95% CI	P-value
Age, ≥75 years	0.795	0.515-1.221	0.296	0.947	0.568-1.578	0.835
Male gender	1.593	1.035-2.455	0.034	1.462	0.914-2.341	0.113
Obesity	1.959	1.268-3.027	0.002	2.050	1.278-3.301	0.003
Diabetes mellitus	1.221	0.792-1.896	0.367	0.909	0.557-1.485	0.703
Dyslipidemia	1.359	0.888-2.087	0.158	1.433	0.862-2.397	0.165
Cerebrovascular disease	0.727	0.442-1.207	0.215	0.643	0.373-1.112	0.114
Ischemic heart disease	0.884	0.550-1.433	0.612	0.873	0.502-1.531	0.634
Heart failure	0.446	0.288-0.688	<0.001	0.419	0.257-0.676	<0.001
eGFR (mL/min/1.73 m^2^)	1.002	0.991-1.013	0.752	0.997	0.985-1.009	0.613

Abbreviations: CI, confidence interval; eGFR, estimated glomerular filtration rate

**Table 5.** Logistic Regression Model of Factors Associated with Nonachievement of Target Blood Pressure Control (≥130/80 mmHg) in Patients without Atrial Fibrillation on Antithrombotic Therapy.

	Univariate model			Multivariate model		
Variable	Odds ratio	95% CI	P-value	Odds ratio	95% CI	P-value
Age, ≥75 years	0.824	0.672-1.008	0.060	0.945	0.751-1.189	0.631
Male gender	1.224	0.998-1.499	0.052	1.225	0.981-1.528	0.073
Obesity	1.668	1.363-2.042	<0.001	1.638	1.319-2.036	<0.001
Diabetes mellitus	0.978	0.800-1.196	0.830	0.919	0.737-1.145	0.451
Dyslipidemia	1.148	0.909-1.446	0.245	1.174	0.905-1.520	0.224
Cerebrovascular disease	0.971	0.794-1.187	0.774	0.755	0.589-0.966	0.025
Ischemic heart disease	0.773	0.631-0.947	0.013	0.717	0.554-0.927	0.011
Heart failure	0.519	0.399-0.677	<0.001	0.517	0.387-0.691	<0.001
eGFR (mL/min/1.73 m^2^)	1.004	0.999-1.009	0.099	1.003	0.998-1.008	0.281

Abbreviations: CI, confidence interval; eGFR, estimated glomerular filtration rate

## Discussion

In this study, most patients on antithrombotic therapy achieved a BP value of <140/90 mmHg, while only approximately 30% of them attained the target BP value of <130/80 mmHg. Univariate and multivariate logistic regression modeling suggested that in patients on antithrombotic therapy, obesity is a modifiable risk factor, whereas comorbidities with cardiovascular diseases, such as heart failure, are negative factors contributing to nonachievement of the target BP.

Although antithrombotic therapy is widely used for the secondary prevention of ischemic stroke, ischemic heart disease, and other cardiovascular diseases and oral anticoagulants have been prescribed to prevent cardiogenic embolism and deep venous thrombosis ^(1), (2), (3)^, antithrombotic therapy is associated with a modest increase in hemorrhagic complications, especially intracranial hemorrhage ^(1), (2), (3)^. The Perindopril Protection Against Recurrent Stroke Study involving patients on antithrombotic therapy demonstrated that antihypertensive drug treatment decreased the mean BP and that the antihypertensive drug group had a 46% lower incidence of intracranial hemorrhage than the placebo group ^(8)^. The Bleeding with Antithrombotic Therapy study found a significant positive correlation between on-treatment BP and the incidence of intracranial hemorrhage, with a cutoff BP value of 130/81 mmHg predicting the onset of intracranial hemorrhage ^(4)^. In the Secondary Prevention of Small Subcortical Strokes trial, which compared patients with lacunar infarction who received aspirin alone with those on dual antiplatelet therapy, the rate of intracerebral hemorrhage in patients with a target SBP value of <130 mmHg was significantly reduced by 67% compared with that in patients with a target SBP value of 130-149 mmHg ^(9)^. The JSH 2019 guidelines state that BP control should be more carefully evaluated in patients with hypertension on antithrombotic therapy with a target BP value of <130/80 mmHg, despite the insufficient evidence for target BP values that prevent bleeding complications during antithrombotic therapy ^(15)^. We recently demonstrated a target BP value of <130/80 mmHg in 31.1% of patients with hypertension on antithrombotic therapy ^(16)^, a low rate of achieving the target BP. Other previous investigations on the association between BP and the hemorrhagic complications of antithrombotic therapy involved not only patients with hypertension but also those without ^(4), (5), (6), (7), (8), (9), (10), (11), (12), (17), (18)^. In this present study, we extended the inclusion criteria of our previous study and investigated the status of BP control and its correlates in patients on antithrombotic therapy. We confirmed that the rate of BP < 130/80 mmHg has remained low and found that more than 90% of the patients were in cardiovascular risk category 3 of JSH 2019. Thus, this population requires stricter BP control. Furthermore, comorbidity with obesity is a modifiable factor associated with nonachievement of target BP. Regarding the BP-lowering effects of weight reduction, it has been estimated that a weight loss of 1.0 kg lowers both SBP and DBP by approximately 1.0 mmHg ^(19)^, and a meta-analysis reported a significant reduction in BP following a weight loss of 4 kg ^(20)^.

Hypertension is the most important modifiable risk factor for the development of cardiovascular diseases such as heart failure ^(21), (22)^. RCTs have demonstrated that the management of hypertension reduces the risk of heart failure by ~50% ^(23), (24)^. However, not only higher BP but also lower BP were associated with higher mortality in patients with heart failure ^(25), (26)^. Thus, the effect of optimal BP levels on the prognosis of patients with heart failure remains unclear. No target BP levels have yet been recommended to adjust the dose of the neurohormonal blockade, including angiotensin-converting enzyme inhibitors, angiotensin receptor blockers, beta-blockers, and/or mineralocorticoid receptor blockers ^(27), (28)^. In RCTs, the use of the maximal tolerable dose is recommended irrespective of BP values. Additionally, lower SBP could be associated with a reduced stroke volume in patients with heart failure. In this study, univariate and multivariate logistic analyses indicated that heart failure was negatively but significantly associated with nonachievement of the target BP. The use of neurohormonal blockade of patients with heart failure was higher than that of patients without heart failure (data not shown). These may affect the association between the attainment of the target BP value of <130/80 mmHg and heart failure in this population.

A meta-analysis by the Blood Pressure Lowering Treatment Trialists’ Collaboration has shown that antihypertensive treatment reduces coronary artery disease in patients with hypertension, regardless of the type of antihypertensive medications ^(29)^. In a meta-analysis of patients with coronary artery disease, target SBP value of <130/80 mmHg was related to a 30% reduction in heart failure and a 20% reduction in stroke, without increasing all-cause mortality or cardiovascular mortality, compared with target SBP value of 136-140 mmHg ^(30)^. Additionally, in a meta-analysis of patients with coronary artery disease without complications of hypertension, antihypertensive treatment reduced incidence of stroke by 23%, myocardial infarction by 20%, heart failure by 29%, cardiovascular death by 17%, and all-cause mortality by 13% ^(31)^. JSH 2019 recommends an antihypertensive target of <130/80 mmHg for patients with coronary artery disease ^(15)^. In this study, univariate and multivariate logistic analyses also indicated that ischemic heart disease was negatively associated with nonachievement of the target BP. Although the precise reason(s) for ischemic heart disease being a negative factor for attaining the target BP value of <130/80 mmHg remain unclear, the number of antihypertensive medications of patients with ischemic heart disease was higher than that of patients without ischemic heart disease (data not shown).

In patients with AF on antithrombotic therapy, insufficient BP control is known to be a major risk factor for hemorrhagic complications ^(4), (5), (6), (7), (8), (9), (10), (11), (12), (15)^. The Japanese Rhythm Management Trial for AF demonstrated that the highest quartile of on-treatment SBP (≥136 mmHg) and SBP at the end of the follow-up period were reported to be significantly associated with the incidence of major hemorrhage and thromboembolism ^(6)^. A recent meta-analysis regarding the BP thresholds for patients with AF on direct oral anticoagulant therapy demonstrated that the nadir SBP for cardiovascular mortality was 130 mmHg ^(32)^. Additionally, worse BP control during follow-up appeared to be a predictor of higher risk of stroke and systemic embolism in patients with AF on antithrombotic therapy ^(33)^. In this study, we also demonstrated that in patients with AF on antithrombotic therapy, obesity was a positive correlate, whereas heart failure was a negative correlate of nonachievement of the target BP. Barrios et al. investigated the BP control rates of anticoagulated patients with hypertension and AF ^(34)^. The target BP values were defined according to the 2013 European Society of Hypertension/European Society of Cardiology guidelines ^(35)^. The authors reported that more than 75% of patients with hypertension with AF attained target BP values ^(34)^. However, the target BP values of the 2013 European guidelines ^(35)^ were different from those of current guidelines ^(13), (14), (15)^. The JSH 2019 proposes that, in addition to adequate antithrombotic therapy and heart rate control, BP control with a target SBP of <130 mmHg should be performed in patients with AF ^(15)^. We conducted a subgroup analysis of the status of BP control in 381 patients with AF as a complication. Only approximately 40% of them attained the target SBP value of <130 mmHg.

The current study has several limitations. First, this is a single-center study, which hinders the generalizability of our findings to the entire population of people on antithrombotic therapy. Second, this was a retrospective study; therefore, the results do not prove a causal relationship between BP and the correlative factors. Third, office BP was measured once for each patient, and the times and methods of measurement were not uniform for all patients. Fourth, the choice of the treatment method was left to the discretion of the attending physician. Finally, the adherence of patients to medication and the effects of lifestyle modifications or nonpharmacological therapy on BP were not considered. Since these limitations may affect the status of BP control and its correlates, careful consideration is needed when comparing the present results to those of other studies.

### Conclusions

In patients on antithrombotic therapy in real-world practice, only ~30% achieved the target BP value of <130/80 mmHg. This study also pointed out that in patients on antithrombotic therapy, obesity was a modifiable factor but cardiovascular comorbidities were negative factors associated with nonachievement of the target BP.

## Article Information

### Conflicts of Interest

None

### Acknowledgement

The authors would like to thank Enago (www.enago.com) for the English language review.

### Author Contributions

All authors contributed to the study conception and design. N.O. is the corresponding author and performed the data collection, made substantial contributions to the analysis and interpretation of the data and wrote this manuscript. A.S. supported the analysis of the data and supervised the manuscript preparation. All authors read and approved the final manuscript.

### Approval by Institutional Review Board (IRB)

Human research ethics committee of Keiaikai (Approval No. 2021001)

## References

[1] Hart RG, Tonarelli SB, Pearce LA. Avoiding central nervous system bleeding during antithrombotic therapy: recent data and ideas. Stroke. 2005;36(7):1588-93. 15947271 10.1161/01.STR.0000170642.39876.f2

[2] Dentali F, Douketis JD, Lim W, et al. Combined aspirin-oral anticoagulant therapy compared with oral anticoagulant therapy alone among patients at risk for cardiovascular disease: a meta-analysis of randomized trials. Arch Intern Med. 2007;167(2):117-24. 17242311 10.1001/archinte.167.2.117

[3] Antithrombotic Trialists’ (ATT) Collaboration, Baigent C, Blackwell L, et al. Aspirin in the primary and secondary prevention of vascular disease: collaborative meta-analysis of individual participant data from randomised trials. Lancet. 2009;373(9678):1849-60. 19482214 10.1016/S0140-6736(09)60503-1 PMC2715005

[4] Toyoda K, Yasaka M, Uchiyama S, et al. Blood pressure levels and bleeding events during antithrombotic therapy: the bleeding with antithrombotic therapy (BAT) study. Stroke. 2010;41(7):1440-4. 20489173 10.1161/STROKEAHA.110.580506

[5] Kai H, Kohro T, Fukuda K, et al. Impact of systolic blood pressure on hemorrhagic stroke in patients with coronary artery disease during anti-platelet therapy: the Japanese coronary artery disease (JCAD) study. Int J Cardiol. 2016;224:112-3. 27648978 10.1016/j.ijcard.2016.09.004

[6] Kodani E, Atarashi H, Inoue H, et al. Impact of blood pressure control on thromboembolism and major hemorrhage in patients with nonvalvular atrial fibrillation: a subanalysis of the J-RHYTHM registry. Am Heart Assoc. 2016;5(9):e004075. 10.1161/JAHA.116.004075 PMC5079049 27620886

[7] Ishii M, Ogawa H, Unoki T, et al. Relationship of hypertension and systolic blood pressure with the risk of stroke or bleeding in patients with atrial fibrillation: the Fushimi AF registry. Am J Hypertens. 2017;30(11):1073-82. 28575205 10.1093/ajh/hpx094

[8] Arima H, Anderson C, Omae T, et al. Effects of blood pressure lowering on intracranial and extracranial bleeding in patients on antithrombotic therapy: the PROGRESS trial. Stroke. 2012;43(6):1675-7. 22535269 10.1161/STROKEAHA.112.651448

[9] Benavente OR, Coffey CS, Conwit R, et al. Blood-pressure targets in patients with recent lacunar stroke: the SPS3 randomised trial. Lancet. 2013;382(9891):507-15. 23726159 10.1016/S0140-6736(13)60852-1 PMC3979302

[10] Uchiyama S, Shinohara Y, Katayama Y, et al. Benefit of cilostazol in patients with high risk of bleeding: subanalysis of cilostazol stroke prevention study 2. Cerebrovasc Dis. 2014;37(4):296-303. 24820203 10.1159/000360811

[11] Nagarakanti R, Wallentin L, Noack H, et al. Comparison of characteristics and outcomes of dabigatran versus warfarin in hypertensive patients with atrial fibrillation (from the RE-LY trial). Am J Cardiol. 2015;116(8):1204-9. 26282726 10.1016/j.amjcard.2015.07.032

[12] Rao MP, Halvorsen S, Wojdyla D, et al. Blood pressure control and risk of stroke or systemic embolism in patients with atrial fibrillation: results from the apixaban for reduction in stroke and other thromboembolic events in atrial fibrillation (ARISTOTLE) trial. J Am Heart Assoc. 2015;4(12):e002015. 26627878 10.1161/JAHA.115.002015 PMC4845276

[13] Whelton PK, Carey RM, Aronow WS, et al. 2017 ACC/AHA/AAPA/ABC/ACPM/AGS/APhA/ASH/ASPC/NMA/PCNA guideline for the prevention, detection, evaluation, and management of high blood pressure in adults: a report of the American College of Cardiology/American Heart Association Task Force on Clinical Practice guidelines. Hypertension. 2018;71(6):e13-5. 29133356 10.1161/HYP.0000000000000065

[14] Williams B, Mancia G, Spiering W, et al. 2018 ESC/ESH guidelines for the management of arterial hypertension: the task force for the management of arterial hypertension of the European Society of Cardiology and the European Society of Hypertension: the task force for the management of arterial hypertension of the European Society of Cardiology and the European Society of Hypertension. J Hypertens. 2018;36(10):1953-2041. 30234752 10.1097/HJH.0000000000001940

[15] Umemura S, Arima H, Arima S, et al. The Japanese Society of Hypertension guidelines for the management of hypertension (JSH 2019). Hypertens Res. 2019;42(9):1235-481. 31375757 10.1038/s41440-019-0284-9

[16] Odaguchi N, Sakima A, Nakada S. Investigation of blood pressure control in hypertensive patients with and without antithrombotic therapy in a real-world setting. Clin Exp Hypertens. 2021;43(3):263-9. 33356616 10.1080/10641963.2020.1860079

[17] Lip GY, Frison L, Grind M. Effect of hypertension on anticoagulated patients with atrial fibrillation. Eur Heart J. 2007;28(6):752-9. 17289744 10.1093/eurheartj/ehl504

[18] Vemulapalli S, Hellkamp AS, Jones WS, et al. Blood pressure control and stroke or bleeding risk in anticoagulated patients with atrial fibrillation: results from the ROCKET AF trial. Am Heart J. 2016;178:74-84. 27502854 10.1016/j.ahj.2016.05.001

[19] Neter JE, Stam BE, Kok FJ, et al. Influence of weight reduction on blood pressure: a meta-analysis of randomized controlled trials. Hypertension. 2003;42(5):878-84. 12975389 10.1161/01.HYP.0000094221.86888.AE

[20] Semlitsch T, Jeitler K, Berghold A, et al. Long-term effects of weight-reducing diets in people with hypertension. Cochrane Database Syst Rev. 2016;3:CD008274. 26934541 10.1002/14651858.CD008274.pub3 PMC7154764

[21] Levy D, Larson MG, Vasan RS, et al. The progression from hypertension to congestive heart failure. JAMA. 1996;275(20):1557-62. 8622246

[22] Bui AL, Horwich TB, Fonarow GC. Epidemiology and risk profile of heart failure. Nat Rev Cardiol. 2011;8(1):30-41. 21060326 10.1038/nrcardio.2010.165 PMC3033496

[23] Kostis JB, Davis BR, Cutler J, et al. Prevention of heart failure by antihypertensive drug treatment in older persons with isolated systolic hypertension. SHEP Cooperative Research Group. JAMA. 1997;278(3):212-6. 9218667

[24] SPRINT Research Group, Wright JT Jr, Williamson JD, et al. A randomized trial of intensive versus standard blood-pressure control. N Engl J Med. 2015;373(22):2103-16. 26551272 10.1056/NEJMoa1511939 PMC4689591

[25] Lee TT, Chen J, Cohen DJ, et al. The association between blood pressure and mortality in patients with heart failure. Am Heart J. 2006;151(1):76-83. 16368295 10.1016/j.ahj.2005.03.009

[26] Lee SE, Lee HY, Cho HJ, et al. Reverse J-curve relationship between on-treatment blood pressure and mortality in patients with heart failure. JACC Heart Fail. 2017;5(11):810-9. 29096790 10.1016/j.jchf.2017.08.015

[27] Ponikowski P, Voors AA, Anker SD, et al. 2016 ESC guidelines for the diagnosis and treatment of acute and chronic heart failure: the task force for the diagnosis and treatment of acute and chronic heart failure of the European Society of Cardiology (ESC) developed with the special contribution of the Heart Failure Association (HFA) of the ESC. Eur Heart J. 2016;37(27):2129-200. 27206819 10.1093/eurheartj/ehw128

[28] Yancy CW, Jessup M, Bozkurt B, et al. 2013 ACCF/AHA guideline for the management of heart failure: executive summary: a report of the American College of Cardiology Foundation/American Heart Association Task Force on Practice Guidelines. Circulation. 2013;128(16):1810-52. 23741057 10.1161/CIR.0b013e31829e8807

[29] Turnbull F; Blood Pressure Lowering Treatment Trialists’ Collaboration. Effects of different blood-pressure-lowering regimens on major cardiovascular events: results of prospectively-designed overviews of randomised trials. Lancet. 2003;362(9395):1527-35. 14615107 10.1016/s0140-6736(03)14739-3

[30] Bangalore S, Kumar S, Volodarskiy A, et al. Blood pressure targets in patients with coronary artery disease: observations from traditional and Bayesian random effects meta-analysis of randomised trials. Heart. 2013;99(9):601-13. 22914531 10.1136/heartjnl-2012-301968

[31] Thompson AM, Hu T, Eshelbrenner CL, et al. Antihypertensive treatment and secondary prevention of cardiovascular disease events among persons without hypertension: a meta-analysis. JAMA. 2011;305(9):913-22. 21364140 10.1001/jama.2011.250 PMC4313888

[32] Minhas JS, Coles B, Mistri AK, et al. What is the optimal blood pressure level for patients with atrial fibrillation treated with direct oral anticoagulants? J Hypertens. 2020;38(9):1820-8. 32453015 10.1097/HJH.0000000000002487

[33] Kollias A, Kyriakoulis KG, Stambolliu E, et al. Prognostic value of office blood pressure measurement in patients with atrial fibrillation on anticoagulation therapy: systematic review and meta-analysis. J Hypertens. 2020;38(1):13-20. 31652181 10.1097/HJH.0000000000002244

[34] Barrios V, Escobar C, Prieto Valiente L, et al. Blood pressure control in anticoagulated patients with hypertension and atrial fibrillation. Blood Press. 2017;26(5):279-83. 28385080 10.1080/08037051.2017.1313094

[35] Mancia G, Fagard R, Narkiewicz K, et al. 2013 ESH/ESC guidelines for the management of arterial hypertension: the task force for the management of arterial hypertension of the European Society of Hypertension (ESH) and of the European Society of Cardiology (ESC). J Hypertens. 2013;31(7):1281-357. 23817082 10.1097/01.hjh.0000431740.32696.cc

